# A novel approach for detecting *Salmonella enterica* strains frequently attributed to human illness—development and validation of the highly pathogenic *Salmonella* (HPS) multiplex PCR assay

**DOI:** 10.3389/fmicb.2024.1504621

**Published:** 2025-01-07

**Authors:** Dayna M. Harhay, Kerry D. Brader, Tatum S. Katz, Gregory P. Harhay, James L. Bono, Joseph M. Bosilevac, Tommy L. Wheeler

**Affiliations:** Roman L. Hruska, US Meat Animal Research Center, Meat Safety and Quality Research Unit, USDA ARS, Clay Center, NE, United States

**Keywords:** non-typhoidal *Salmonella enterica*, highly pathogenic *Salmonella* (HPS), multiplex PCR, secreted effector, fimbriae, serovars of concern (SoC)

## Abstract

**Introduction:**

Non-typhoidal *Salmonella enterica* (NTS) are leading bacterial agents of foodborne illnesses and a global concern for human health. While there are over 2,600 different serovars of NTS, epidemiological data suggests that certain serovars are better at causing disease than others, resulting in the majority of reported human illnesses in the United States. To improve food safety, there is a need to rapidly detect these more pathogenic serovars to facilitate their removal from the food supply.

**Methods:**

Addressing this need, we conducted a comparative analysis of 23 closed *Salmonella* genomic sequences of five serotypes. The analysis pinpointed eight genes (*sseK2*, *sseK3*, *gtgA/gogA*, *avrA*, *lpfB, SspH2, spvD,* and *invA*) that in combination, identify 7 of the 10 leading *Salmonella* serovars attributed to human illnesses in the US each year (i.e., Serovars of Concern or SoC). A multiplex PCR assay was developed to detect the presence of these genes, with strains amplifying five or more targets designated Highly Pathogenic *Salmonella*, or HPS. The utility of the resulting HPS assay for identifying SoC was examined *in silico*, using BLAST to determine the distribution of gene targets among closed *Salmonella* genome sequences in GenBank (n = 2,192 representing 148 serotypes) and by assaying 1,303 *Salmonella* (69 serotypes), isolated from FSIS regulatory samples.

**Results and discussion:**

Comparison of serotypes identified by the assay as HPS, with those identified as SoC, produced an Area Under the Curve (AUC) of 92.2% with a specificity of 96% and a positive predictive value of 97.4%, indicating the HPS assay has strong ability to identify SoC. The data presented lay the groundwork for development of rapid commercial assays for the detection of SoC.

## 1 Introduction

Non-typhoidal *Salmonella enterica* (NTS) are the leading cause of foodborne illness attributed to bacteria in the United States, Canada, and Mexico (Scallan et al., 2011; National Enteric Surveillance Program, 2022; Godínez-Oviedo et al., 2020) and the second most common causative agent among reported zoonotic infections in the European Union (EU) (EFSA and ECDC, 2021). NTS are also the primary cause of deaths attributed to foodborne disease around the world [59,771 in 2017 (Stanaway et al., 2019)]. The success of NTS as human pathogens is attributed to their genotypic and phenotypic diversity. Composed of over 2,600 serotypes (Issenhuth-Jeanjean et al., 2014), some NTS are adapted to particular hosts or environments (Merkushova et al., 2023), while others are generalists, capable of persisting in a variety of hosts or environments (Waldner et al., 2012; Jechalke et al., 2019). In production agriculture, certain NTS serovars are noted for causing clinical illnesses in animals, but more often they are harbored as part of the animal’s normal flora and do not cause disease (Kuria, 2024). As a result, it is not uncommon to isolate *Salmonella* from healthy animals on farm or at harvest (Brichta-Harhay et al., 2008; Kunze et al., 2008; Loneragan et al., 2012; Schmidt et al., 2012; Gutema et al., 2019).

Comparisons of NTS serotypes isolated from animals in pre- and post-harvest settings have revealed a disparity, in that those commonly isolated from animals and meat products do not necessarily reflect those most attributed to meat and poultry related outbreaks and illnesses (Bosilevac et al., 2009; Brichta-Harhay et al., 2011; Schmidt et al., 2012; Cheng et al., 2019). An example of this can be seen with NTS isolated from ground beef, where surveys show serotypes Montevideo and Anatum are predominantly isolated, while serotypes Newport and Typhimurium are observed much less frequently (Bosilevac et al., 2009). Despite being rarely observed as contaminants, Newport and Typhimurium have been attributed to 17 ground beef related outbreaks (Centers for Disease Control and Prevention (CDC), National Outbreak Reporting System (NORS) data 2009–2018), resulting in 930 illnesses and one death, while Montevideo has been attributed to just two ground beef related outbreaks over the same time period, resulting in 124 illnesses and no deaths, and Anatum has not been attributed to any ground beef related outbreaks (Centers for Disease Control and Prevention, 2023). Data on serotypes attributed to ground beef outbreaks was obtained from CDC’s NORS dashboard; accessed 8/31/2023. That site is no longer available as it has become part of the new BEAM dashboard.

Historically however, all NTS have been regarded as human pathogens, irrespective of serotype, because of their ability to invade intestinal epithelial cells and potential for causing systemic disease (Groisman and Ochman, 1997; Hensel, 2000). Yet, the past 30 years of surveillance data have also demonstrated that certain serovars consistently contribute to most human illnesses year over year (Collins et al., 2022), with ~10% of serotypes isolated each year (43/442) attributed to ~90% of human illness isolates characterized (n = 45,515) (Centers for Disease Control and Prevention, 2022). This observation has fueled the idea that to improve human health outcomes with regard to *Salmonella* infections, the food safety community should focus control efforts by specifically targeting serovars that pose a greater risk to human health (i.e., Serovars of Concern, or SoC) rather than attempting to broadly manage all *Salmonella* contamination (United States Department of Agriculture Food Safety and Inspection Service, 2024). Along these lines, two recent studies have described methods for analyzing *Salmonella* outbreak data, to identify which serovars attributed to meat and poultry contribute to most human illnesses in the US, year over year (Katz et al., 2024; Marshall et al., 2024). The study by Katz et al., identified a list of 21 SoC, while the study by Marshall et al., described 13 serovars associated with a high burden of outbreak illnesses. The resulting serovar lists overlapped substantially suggesting there may be genetic similarities between these more pathogenic versions of NTS.

The increase in *Salmonella* genome sequence data in recent years has provided the opportunity to characterize, through comparative genomics, the NTS virulence gene repertoire and several studies have provided summaries to this effect (den Bakker et al., 2011; Worley et al., 2018; Rakov et al., 2019; Wang et al., 2020). Furthermore, Fenske and colleagues recently reported an analysis of the distribution of 182 virulence genes among high and low illness incidence serovars and found that NTS associated with a higher incidence of human illness appear to possess a common repertoire of virulence genes, that likely enable them to more readily cause disease, possibly at a lower infectious dose (Fenske et al., 2023). These seminal investigations have provided a broad view of the NTS virulence gene landscape and insight into the differences within and between serovars that influence host pathogen interactions. What has been lacking to date, however, is the identification of a limited NTS virulence gene set that can be harnessed for the rapid detection of the more pathogenic versions of *Salmonella* (i.e., Highly Pathogenic *Salmonella* or HPS).

In Europe, poultry production control programs targeting the leading *Salmonella* serovars (Enteritidis, Typhimurium and the monophasic version of Typhimurium, I,4,[5],12:i:-; also known as SE/ST serovars) have been implemented^1^ with the goal of reducing human illnesses by restricting the presence of these *Salmonella* serovars. Accordingly, real-time PCR assays have been developed for the identification of these serovars (Maurischat et al., 2015; Zhengwei et al., 2023) and commercial versions of rapid assays for identifying SE/ST are available. However, these leading serovars account for only ~35% of the US human illness isolates characterized by CDC each year (CDC BEAM Dashboard,^2^ accessed September 20, 2024). Furthermore, studies show this targeted serovar approach has stalled out in recent years, and there is a perceived need for a more proactive screening approach based on the presence of particular virulence genes that contribute to overall pathogenicity of *Salmonella*, irrespective of serovar, as opposed to a reactive approach of targeting particular serovars, based on outbreak data or trends in serovar prevalence in food commodities (Petrin et al., 2023).

To assist with this effort, we conducted a comparative genomics analysis of 23 closed genome sequences of *Salmonella* serovars that are frequently associated with human illnesses (serovars Typhimurium, Newport and Dublin) with serovars that are less frequently associated with human illness (Montevideo and Anatum), that had been isolated from cattle and beef products or humans. Analysis of this test set of carefully selected closed genome sequences led to the identification of 8 virulence genes (*sseK2*, *sseK3*, *gtgA/gogA*, *avrA*, *lpfB, SspH2, spvD,* and *invA*) that tended to be present in the genomes of serovars more commonly associated with human illness. Here were report on the utility of these 8 virulence genes for identifying *Salmonella* SoC. A multiplex PCR assay targeting the detection of these 8 genes was developed (designated the HPS assay) and evaluated using 1,303 *Salmonella* strains across 69 different serotypes. Further, the DNA sequence of each target amplicon was subjected to BLAST analysis (BLAST: Basic Local Alignment Search Tool, n.d.) to examine the distribution of hits among 2,192 closed *Salmonella* genome sequences of 148 serovars present in the NIH sequence database, GenBank. The overall performance of the HPS assay for identifying *Salmonella* SoC was evaluated and its strengths and limitations are presented and discussed.

## 2 Materials and methods

### 2.1 HPS target identification and multiplex PCR assay development

Comparative genomic analysis of 23 *Salmonella enterica* ssp. *enterica* closed genome sequences (listed in Table 1) was conducted using Geneious v.11.1^3^ Mauve plugin; progressiveMauve algorithm, with the following settings: Automatically calculate seed weight; Match seed weight = 15; Minimum LCB Score = 30,000; Compute Locally Collinear Blocks (LCBs); Full alignment; Gapped Aligner = MUSCLE 3.6 (Darling et al., 2004). Prior to Mauve analysis, care was taken to ensure that all linearized genome sequences had the same +1 position as that of the *S. typhimurium* SL1344 origin of replication, identified using OriFinder (Gao and Zhang, 2008). Plasmid sequences (n = 16) were grouped by size and analyzed using Mauve. The largest of the resulting 187 ‘locally collinear blocks’ (LCBs) (n = 42 LCB ranging from 10 to 784 kbp) were visually inspected for differential regions of homology. Genes that demonstrated greatest homology among serovars commonly attributed to human illness were analyzed with Geneious Primer Design, which is a modified version of Primer3 v.2.3.7, with the goal of producing PCR amplicons ranging in size from 844 bp to 275 bp (amplicon sizes listed in Table 2). The resulting primer sequences, genes targeted, binding locations, and annealing temperatures are listed in Table 2. Nucleotide BLAST analysis of predicted amplicons based on the sequences present in *S. typhimurium* strain SL1344, was conducted using BLAST+ 2.8.0. The results of this *in silico* analysis are summarized for the top 55 serovars in Table 3 (full list summarized in Supplementary Table S1) with the percent homology cutoff used to determine gene presence or absence indicated, and the distribution of genes targeted in 2,192 closed *Salmonella* genome sequences of 148 serotypes present in GenBank (data accessed September 12, 2024).

**Table 1:** Description of serotype, genbank accession numbers and sequence types (ST) of genomes used in the Mauve analysis, and heat map of the percent homology (dark blue highest and white lowest) of the HPS gene targets present in each strain, in comparison with *S. typhimurium* SL1344.

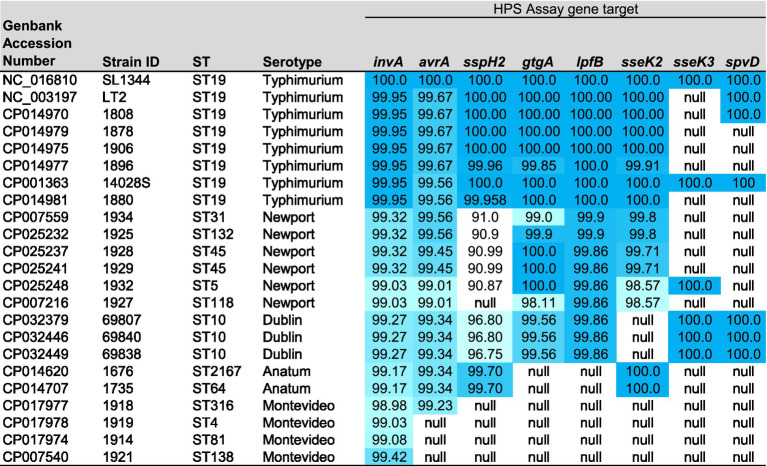

**Table 2:** Description of genes targeted, location on chromosome or in plasmid, role in pathogenesis if known, primers used to identify each gene, amplicon size, and PMID for references describing gene function.

Gene	HPS Assay ID	SL1344_Locus ID	Gene size (bp)	Location	Product	Function	Primer name	HPS primer sequences (5′–3′)	Amplicon size (bp)	Reference (PMID)
*sseK2*	HPS-6	RS10980	1,047	chromosome	Secreted effector. Arginine glycosyltransferase.	Enhances bacterial survival in host by avoiding clearance mechanisms.	HPS-6-F	GCACGTTTTAATGCCGCTTTT	844	32131463
							HPS-6-R	GATGCTCGCTGCGGTTAAC		
*sseK3*	HPS-1	RS10000	1,008	GIFSY-1 or ST64B	NleB homologue, secreted effector shown to inhibit TNF-a and NF-kB host signalling by preventing translocation of immune regulator NF-kB to the host cell nucleus	Interference with host innate immune response; enhances bacterial survival and persistence in host; avoiding host clearance mechanisms	HPS-1-F	TGCCTTCATCAGGTAGTGCAA	739	28069818; 32131463; 32974215; 29449376
							HPS-6-R	GATGCTCGCTGCGGTTAAC		
*avrA*	HPS-3	RS14835	906	SPI-1*	yopJ homologue, secreted effector acetyltransferase; disrupts host innate immune signalling by targeting NF-kB.	Hijacks host epithelial cells, enhancing survival; reverses host inflammatory response; blocks recruitment of phagocytic leukocytes	HPS-3-F	CGAGTTTATCGCCTCAGCCT	663	30025209; 32842467; 21899703
							HPS-3-R	TCGTTGTRCGCCTTGAGTAT		
*lpfB*	HPS-5	RS18760	699	chromosome	PapD-like chaperone.	Long Polar fimbriae (lpf) promotes binding and colonization of M cells located at Peyer’s patch.	HPS-5-F	GCTGGCGTTGATTGCTCAAA	557	33614531; 28630073
							HPS-5-R	TCAATGAGCCTTTTGCCGGA		
*spvD*	HPS-4	RS24010	651	sPV plasmid	Secreted effector. Inhibits NF k-B signalling pathway. Targets p65.	Suppresses host immune response and contributes to systemic/invasive infection.	HPS-4-F	TGGTAGTGCGTCATCCCAAG	452	27232334; 27789710
							HPS-4-R	GAGCTATGTAGTTCTCTGCGCT		
*sspH2*	HPS-7	RS11515	2,367	SPI-12*	Secreted effector. E3-ubiquitin ligase	Alters host innate immune response and enhances bacterial survival in host cells.	HPS-7-F	ACTGTCTGAACGTACTTTGCAG	381	30049795; 33735297
							HPS-7-R	CGGTCCTCGCAGCTTGAG		
*gtgA*	HPS-2	RS05020	687	GIFSY-2	pipA (SPI-5) and gogA (GIFSY-2) homologues, secreted effectors – zinc metaloprotease that targets and cleaves NF-kB transcription factors p65, RelB, cRel	Inhibits host innate immune response and increases ability to infect host	HPS-2-F	CGCCTCCAACATGATGGTCT	338	30049795; 33735297
							HPS-2-R	TCCAGGTTGAGGGCAATCAC		
*invA*	invA	RS14990	2058	SPI-1	part of Type III Secretion System apparatus	Contributes to invasion of gut epithelial cells.	INVA-F	TATCGCCACGTTCGGGCAA	275	1528198; 1624429
							INVA-R	TCGCACCGTCAAAGGAACC		

Locations designated with an asterisk (*) indicate that the gene listed is not always present in that location, due to diveristy in pathogenicity island gene content within and between Salmonella serovars.

**Table 3:** Summary of the in silico analysis of the distribution of HPS genes among 2,192 closed *Salmonella* genome sequences of 148 serotypes present in genbank and the anticipated HPSi, in comparison with serovars observed with HPS analysis of 1,303 *Salmonella* isolated from 1,244 positive enrichment samples obtained from FSIS, ranked from greatest to least contribution to human illness (with gray color scale (darkest to lightest) indicating highest to lowest percent identified by commodity type), as determined by analysing human illness isolate data reported by CDC over a five year window, from 2018 to 2022.

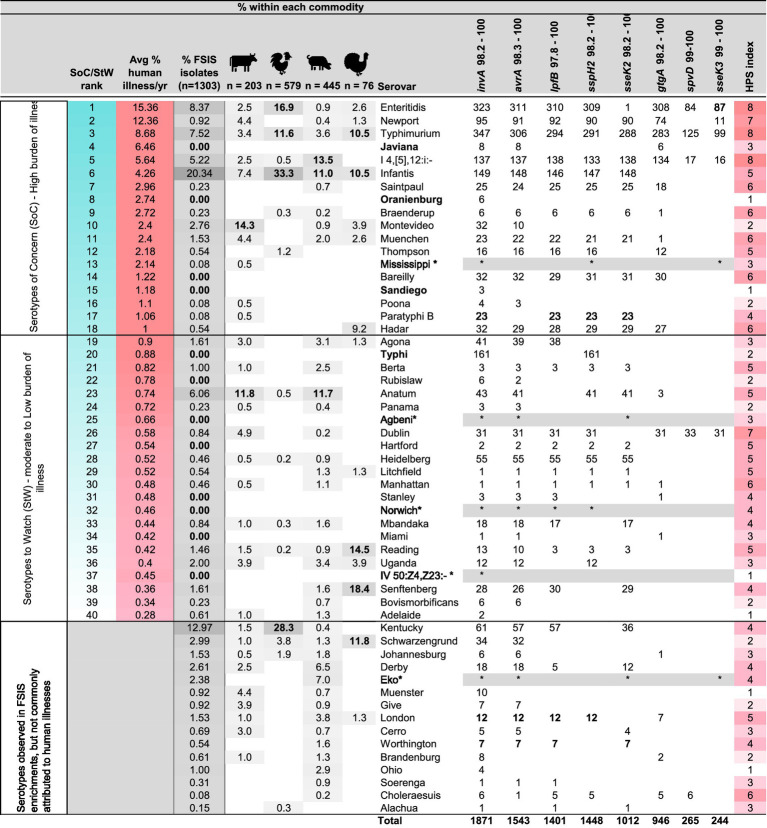

**Figure 1:**
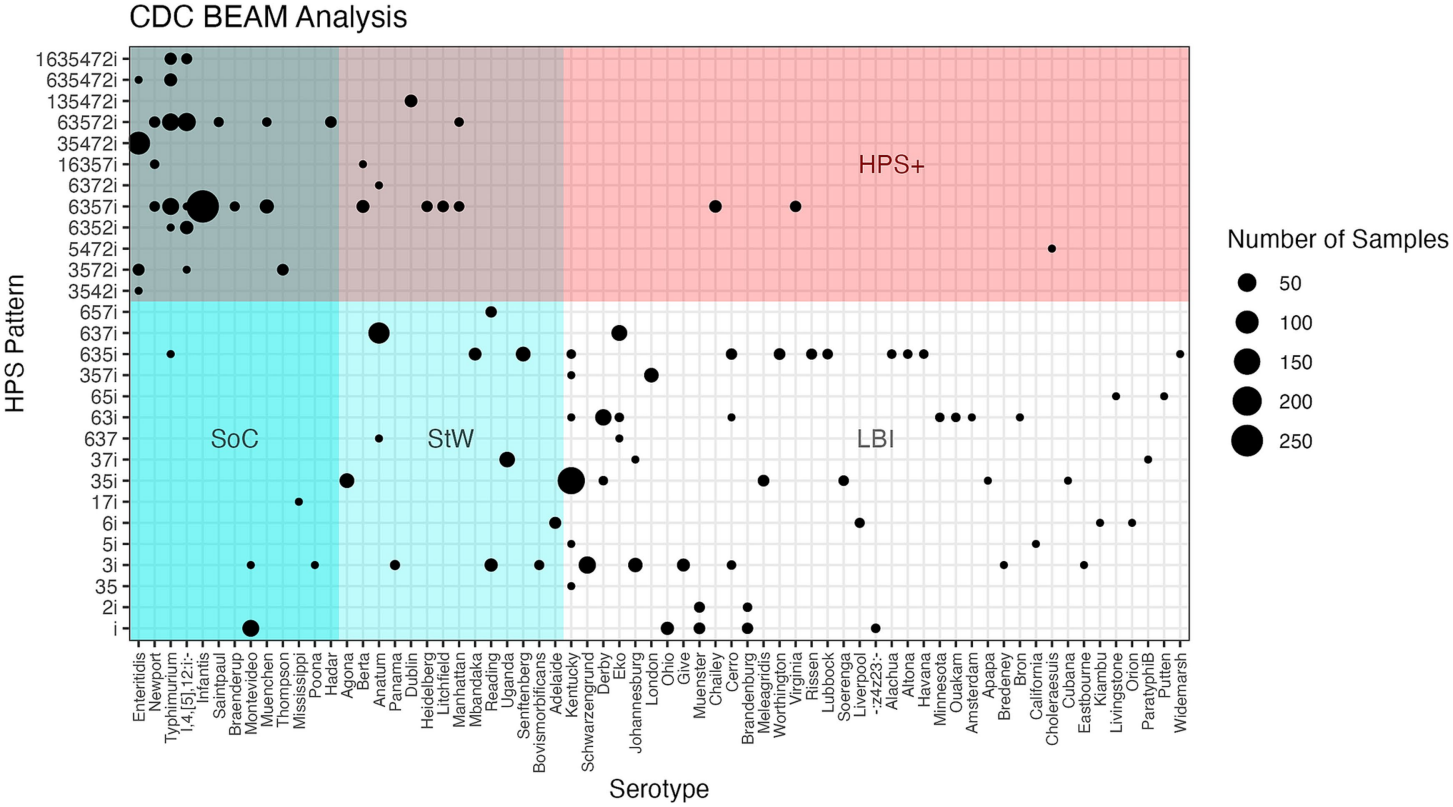
Confusion matrix showing HPS genotypes observed for 1,303 *Salmonella* isolates from FSIS enrichment samples, with the 69 serovars observed ranked from highest to lowest [(Serovar of Concern (SoC) - high burden of illness, Serovar to Watch (StW) - moderate burden of illness, and Low Burden of Illness (LBI)], with bubble size indicating the number of isolates observed for each unique HPSi:serotype combination.

Red and blue color scales correspond with SoC/StW, and HPSi designations in Figure 1.

The protocol for conducting the HPS multiplex PCR assay can be found in Protocols.IO (Harhay et al., 2024), but briefly described, it contained the following in a 25 μL reaction volume: 2.5 U Bullseye H.S. Taq Polymerase (MidSci, BE225108; 5 U/ul), 1 X Buffer II (10 X buffer with 15 mM MgCl_2_), 0.24 mM dNTPs (NEB, N0447L), primers at a final concentration ranging from 0.16 to 0.25 μM, PCR grade water to bring volume to 20 μL, and 5 μL of template DNA. *Salmonella* isolates to be tested with the HPS assay were struck for isolation onto either XLD or Tryptic Soy Agar (TSA) plates, which were incubated at 37°C overnight. Isolated colonies were then sub-cultured to Trypticase Soy Broth (TSB) and incubated at 37°C, static for 18 to 20 h. From the overnight cultures, 5 μL was transferred into 200 μL of prepared BAX System lysis reagent (Hygiena, Camarillo, CA, United States) and lysed according to manufacturer’s protocol (incubation at 37°C for 20 min followed by 95°C for 10 min).

Reaction mixtures were subjected to a modified version of the touch down and extension thermal cycler amplification program previously described [(Paton and Paton, 1998); with specific modification as described in Harhay et al. (2024)]. *S. typhimurium* strain ATCC 14028 produces amplicons of the correct size for all 8 gene targets and was used as a control and DNA ladder for gel electrophoresis as shown in Harhay et al. (2024). Amplicons were visualized by agarose gel electrophoresis and staining with ethidium bromide (Sambrook and Russell, 2006; Lee et al., 2012).

### 2.2 *Salmonella* Isolation from FSIS positive primary enrichments, serotyping, and HPS analysis

*Salmonella*-positive primary enrichment broth samples (n = 1,244) were received from three USDA FSIS laboratories (~415 samples per location) over the course of 1 year, from June 2021 to May 2022 (Supplementary Table S2). Samples were stored as glycerol stocks (16% vol/vol glycerol) at −20°C and shipped to USMARC frozen on dry ice. Upon arrival, samples were thawed at room temperature (~30 min), vortexed, and then 1 mL from each sample was subjected to immunomagnetic separation (IMS) using 20 microliters (20 μL) of Dynal anti-*Salmonella* Dynabeads™ (Applied Biosystems, Foster City, CA, United States) as previously described (Bosilevac et al., 2009). Recovered IMS beads were transferred to 3 mL of RVS medium and incubated at 42°C for 18–22 h.

Post incubation, RVS secondary enrichments were streaked for isolation onto XLD agar (Oxoid Thermo Fisher, Hampshire, United Kingdom) and incubated at 37°C for 20 h. Typical *Salmonella* colonies (pink with a black center and clear outer ring, or rarely, clear colonies with no evidence of H_2_S production) were picked (up to eight putative *Salmonella* per sample) for downstream serovar characterization and HPS genotype profiling with the HPS assay. Two putative *Salmonella* isolates from each sample were cultured in TSB at 37°C for 18–20 h. These cultures were used to make two different DNA lysates, a BAX lysate as described above, and a proteinase-K lysate, made by combining 5 μL of TSB culture with 150 μL of PK-lysis buffer containing 1X Tris-EDTA, pH 8.0 (145 μL) and 5 μL proteinase K (20 mg/mL) per reaction, followed by incubation at 95°C for 15 min. Lysates were stored at −20°C until used as DNA template in three different multiplex PCR reactions to determine *Salmonella* molecular serovar., as previously described (Echeita et al., 2002; Herrera-León et al., 2004, 2007; Harhay et al., 2024). A subset of isolates (~10%) were selected for serovar confirmation using traditional O and H antisera agglutination typing methods. The serotypes and HPS genotypes identified for 1,303 unique isolates are summarized by commodity in Supplementary Table S3.

### 2.3 Examination of *Salmonella* incidence data to identify serotypes most often associated with human illness in the US

To assess the sensitivity and specificity of the HPS Assay for identifying *Salmonella* of greater concern for human health, it was necessary to develop a framework for determining which serotypes fell into this category. While recent studies have presented methods for identifying *Salmonella* serovars of concern (SoC) using analyses of outbreak data (Katz et al., 2024; Marshall et al., 2024), we endeavored to present a list of SoC based on analysis of *Salmonella* isolate data characterized by the CDC, and representative of serotypes associated with both sporadic and outbreak illnesses. This analysis entailed using *Salmonella* serotype data for human-derived isolates collected in the United States from 2018 to 2022, for which we examined the association between serotype and salmonellosis incidence (inferred from the number of isolates CDC characterized and reported) by year. The data assessed included *Salmonella* serotype information for isolates identified in PulseNet, NARMS, NORS (*Salmonella* outbreak data) and Epi Info, coordinated through the System for Enteric Disease Response, Investigation, and Coordination (SEDRIC).

For this analysis, BEAM Dashboard—Report Data (Centers for Disease Control and Prevention, 2022) were downloaded^4^ on January 29, 2024. Analyses were conducted using the R statistical programming language version 4.2.1 (R Core Team, 2023). As illustrated in Supplementary Figure S1, *Salmonella* isolate data from 2018 to 2022 were summarized by serotype to identify the total number of isolates characterized, as well as the number of isolates resulting from multistate outbreaks (MSOB), by year. The percent contribution to the total number of isolates characterized, was then calculated by year. These data were aggregated, resulting in 2,159 datapoints over the five-year window, ranging from 1 to 8,117 isolates per serotype identified. The top 13.7% of data points (ranging from 0.2 to 18.7% contribution per year; n = 297 datapoints and 93% of isolates characterized) were ranked from least to greatest, and a quartile analysis was performed to examine the distribution.

The top quartile (n = 75 data points) was further examined and serotypes (n = 40) that had at least one year with ≥ 10 MSOB isolates, and a total contribution of >0.2% over the five-year window were ranked from greatest to least. Those with an average contribution ≥ 1% (n = 18 serotypes and 76.7% of isolates) were classified as SoC, represent an increased burden of illness, and are summarized in Table 3. The remainder of the list (n = 22 serotypes and 9.7% of isolates) were classified as Serotypes to Watch (StW), as they represent a moderate burden of illness. The overlap between HPS genotype, serovar, SoC/StW, and abundance in the FSIS dataset, is illustrated in Figure 1.

### 2.4 Sensitivity and specificity of the HPS assay for identifying *Salmonella* serotypes of greater concern for human health

**Figure 2:**
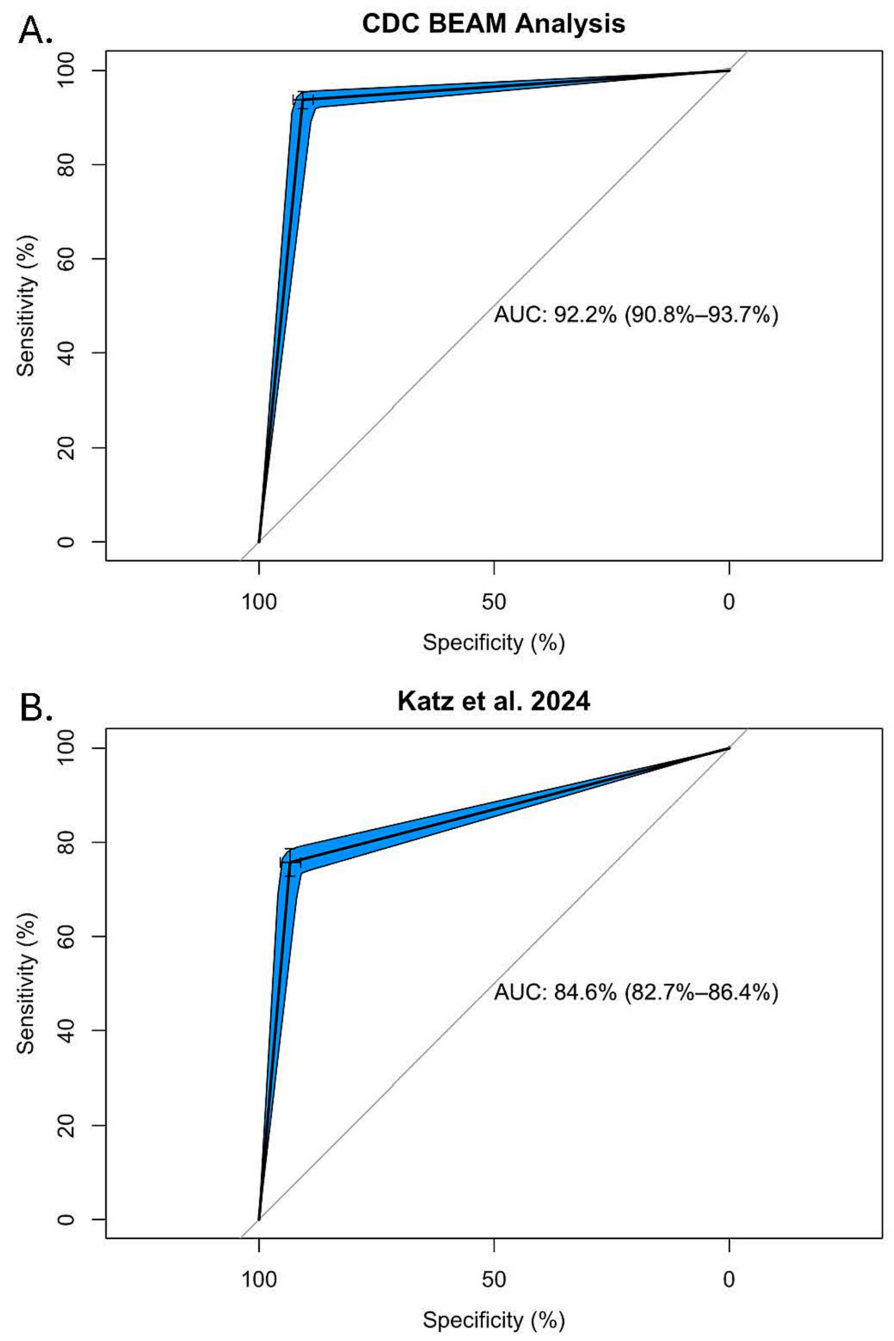
ROC-AUC analysis of the HPS assay for identifying SoC as determined by analysis of isolate data obtained from the CDC’s BEAM Dashboard **(A)**, or the list of SoC identified by Katz et al., with statistical analysis of CDC outbreak data attributed to meat and poultry **(B)**.

Serotypes with an HPSi ≥ 5 (HPS index, i.e., five or more gene targets amplified with the HPS assay) were compared with the list of SoC and StW summarized in Table 3, as well as the list of SoC generated by (Katz et al., 2024). Serotype:HPSi were compared with both SoC lists to ensure that a holistic group of important *Salmonella* serotypes was evaluated. The agreement between HPS and SoC definitions was calculated using confusion matrix statistics, specifically Area Under the Curve (AUC), sensitivity, and specificity (Altman and Bland, 1994; Kuhn, 2008) (Figure 2).

## 3 Results

### 3.1 Comparative genomics and BLAST analysis of genes targeted by the HPS assay

Comparative genomic analysis of 23 closed *Salmonella* genomes and 16 plasmids (Table 1) using Mauve, produced 187 LCB. LCB over 10kbp in size were visually inspected using the Geneious LCB viewer, for regions of greatest homology among serotypes with an increased association with human illness. In all, seven genes (*sseK2*, *sseK3*, *gtgA/gogA*, *avrA*, *lpfB, SspH2,* and *spvD*) were identified as potential targets based on their presence in the genome or plasmid sequences of *Salmonella* serotypes more commonly attributed to human outbreaks and illnesses. Notably, six of the seven genes identified were determined to encode secreted effector proteins that have been shown to alter host signal transduction pathways, and impede host innate immune response [*sseK2*, *sseK3*—(Araujo-Garrido et al., 2020); *gtgA*/*gogA*—(Jennings et al., 2018; Takemura et al., 2021); *avrA*—(Labriola et al., 2018; Yin et al., 2020); *spvD*—(Grabe et al., 2016; Rolhion et al., 2016); *sspH2*—(Miao et al., 1999; De Meyer et al., 2021)]. One of the targets, the *spvD* gene, was found to be on the *spv* virulence plasmid (Guiney et al., 1995), while the other gene targets were located on the chromosome. The seventh gene (*lpfB*) encodes a chaperone protein, important in the synthesis of long polar fimbriea (lpf) which have been shown to promote binding and colonization of *Salmonella* to M (microfold) cells located in the Pyere’s patch region of the ileum in the small intestine (Baümler and Heffron, 1995; Gonzales et al., 2017; Hansmeier et al., 2017). Primer sequences were selected in these genes, as well as primers previously identified for targeting the *Salmonella* specific portion of the *invA* gene [which has been shown to be present in >99.8% of *Salmonella* genome sequences (Rahn et al., 1992; Nucera et al., 2006)], such that a ladder of 8 amplicons ranging in size from 844 to 275 bp is produced and readily visualized with agarose gel electrophoresis (Table 2; Harhay et al., 2024).

BLAST analysis of the predicted amplicons (including primer sequences) showed them to be highly specific for *Salmonella enterica* subsp. *enterica,* with the exception of the *lpfB* amplicon which showed potential for cross-reactivity with a homolog (average amplicon identity of 90.9%) present in certain species of *Enterobacter* (specifically *hormaechei* and *asburiae* sequences present in genbank). The distribution of amplicons among 2,192 closed *Salmonella* genome sequences of 148 serotypes is summarized in Supplementary Table S1, and for the top 55 serovars (n = 1,871 sequences) attributed to human illnesses and or isolated from the FSIS enrichments in Table 3. This *in-silico* analysis confirmed the presence of HPS gene targets in serotypes Typhimurium, Newport and Dublin, but also showed differential presence in other serotypes commonly found in the CDCs list of top 20 *Salmonella* attributed to human illnesses (Centers for Disease Control and Prevention, 2018).

### 3.2 HPS assay results for *Salmonella* isolated from FSIS enrichments

To examine the utility of the HPS assay for identifying *Salmonella* SoC, 1,303 *Salmonella* were isolated from 1,244 positive enrichment samples provided by FSIS over the course of 1 year. These isolates were serotyped and their HPS genotype determined. The serotypes and percent observed from enrichments of each commodity are summarized in Supplementary Table S3 and Figure 1. Five serovars, namely Infantis (20.3%), Enteritidis (8.4%), Typhimurium (7.4%), Schwarzengrund (2.92%), and Reading (1.46%), were the only serovars found in all four meat commodities tested (Table 3). HPS analysis of the FSIS isolates showed 29 different HPS genotypes were observed, ranging from all 8 amplicons (scored as 1635472i observed for 14 Typhimurium and monophasic Typhimurium isolates) to just the amplicon for *invA* (scored as i; observed for 63 isolates of serovars Montevideo, Brandenburg, Muenster, −:z4,z23:-, and Ohio).

Overall, approximately 50% of the isolates from FSIS enrichments showed HPSi ≤ 4 amplicons, while the other 50% showed HPSi ≥ 5. The most common HPS genotype observed was 6357i (29% of isolates) found in 13 serotypes, of which S. infantis was the greatest contributor (264 of 1,303 *Salmonella* isolated). Remarkably, 3 isolates were observed to not amplify the *invA* target (two with HPS genotypes of 637, an Anatum and an Eko isolate from different pork samples; and one isolate with an HPS gene pattern of 35, a Kentucky isolate from a chicken enrichment), although not confirmed with sequence analysis if this was due to the absence of the *invA* gene or differences in primer binding sites. Importantly, analysis of HPSi by serotype showed that seven of the top 10 serovars on the CDC’s list of Top 20 *Salmonella* (Centers for Disease Control and Prevention, 2018) had HPSi ≥ 5. This observation led to a broader examination of HPSi among serovars present in CDC isolate data over a 5-year window (2018–2022).

### 3.3 Analysis of BEAM dashboard isolate data to define a list of SoC

To identify *Salmonella* serovars attributed to the greatest number of illnesses each year, BEAM Dashboard data (from 2018 to 2022) were downloaded and the contribution of 102,384 isolates to human illnesses and multistate outbreaks was assessed. This analysis produced a list of 40 serotypes, attributed to 86.3% of all isolates (a proxy for illnesses) characterized by CDC in that time window (Table 3). The top 18 serotypes of that list were attributed to 76.7% of isolates and given their prominent and repeated attribution to human illness, were classified as SoC. The second half of the list, classified as StW, comprised 22 serovars with moderate but repeated contribution to human illnesses (9.7% of all isolates characterized).

### 3.4 Comparison of SoC/StW classification with serotype:HPSi profiles observed for FSIS isolates

Having identified a list of SoC/StW from CDC isolate data, we applied this ranking to the serotypes isolated from the FSIS samples and used the HPS assay results of these isolates to examine the association between HPSi and SoC/StW. For the 1,303 *Salmonella* strains isolated and serotyped, 864 (66.3%) were serotypes classified as SoC/StW. The remaining 439 (33.7%) were serotypes categorized as being of lesser concern for human health, representing a low burden of illness (LBI). Examining the overlap between SoC/StW and HPSi ≥ 5 showed that 97% (635/652) of isolates with HPSi ≥ 5 were serotypes on the SoC/StW list, again demonstrating a high concordance. Illustrating this further, four of the top five serovars on the SoC list (Enteritidis, Newport, Typhimurium, and monophasic Typhimurium) all had HPSi ≥7. Conversely, 64.8% of isolates with HPSi ≤ 4 (422/651) were serotypes identified as LBI.

Sensitivity and specificity analyses also showed the HPS assay to have moderate-high sensitivity (73.5%) and a very high positive predictive value (97.4%) for isolates with an HPSi ≥ 5 being on the SoC/StW list, demonstrating a very strong ability of the HPS assay to identify SoC/StW. Specificity was found to be 96.3% with a negative predictive value of 64.8%, revealing a slight trend of the HPS assay to miss some serotypes on the SoC/StW lists. There were 13 serovars in this group which was dominated by Anatum (n = 78), Montevideo (n = 36), Uganda (n = 26), Agona, and Senftenberg each with 21 isolates, and Reading (n = 19). Taken together, an HPSi finding of 5 or greater is very strong, albeit slightly conservative evidence of an isolate being a SoC/StW. Figure 1 illustrates the distribution and abundance of FSIS serotype:HPS genotype, with serovars ranked by classification of SoC/StW, or LBI on the x-axis.

Finally, the AUC was calculated to examine the ability of HPSi ≥ 5 to correctly identify SoC/StW. AUC was calculated for two different lists of SoC: the list generated by Katz et al., based on analysis of outbreak data attributed to meat commodities (Katz et al., 2024), and the SoC/StW list generated here using CDC isolate data as a proxy for human illnesses. As shown in Figure 2, HPSi ≥ 5 and both SoC definitions had high concordance, with an AUC of 92.2% for the CDC BEAM isolate SoC/StW list (Figure 2A), and 84.6% for the list of 21 SoC identified from outbreak analyses (Figure 2B).

## 4 Discussion

Historically, differences in *Salmonella* pathogenicity have been under appreciated and it was generally accepted that all serovars were equally capable of making humans sick. However, genomic sequencing efforts over the past two decades and subsequent comparative genomic analyses have revealed substantial differences in virulence gene content both among and within serovars (den Bakker et al., 2011; Suez et al., 2013; Worley et al., 2018; Cheng et al., 2019; Rakov et al., 2019; Fenske et al., 2023). These findings, in combination with an increased understanding that a relatively small subset of serovars contribute to the majority of illnesses, have resulted in a shift in the approach being considered for controlling *Salmonella*, from prevalence-based methods to techniques that assess both contamination level and pathogenicity level of the contaminating serovar [United States Department of Agriculture Food Safety and Inspection Service, 2022; National Advisory Committee on Microbiological Criteria in Foods (NACMCF), 2024], with the goal of removing products contaminated with higher levels of *Salmonella* or with serovars of greater significance for human health (i.e., SoC).

With this new approach, FSIS aims to improve public health by reducing SoC in the products they regulate, which the Interagency Food Safety Analytics Collaboration (IFSAC) estimates contribute to 38% of foodborne *Salmonella* illnesses annually (United States Department of Agriculture Food Safety and Inspection Service, 2020). It is anticipated that successful implementation of this approach will help the U.S. reach the Healthy People 2030 goal of reducing *Salmonella* infection incidence from 15.3 cases per 100,000 to 11.5 per 100,000 (Office of Disease Prevention and Health Promotion, 2023). For this to work however, new testing methods are needed for the rapid identification of more pathogenic versions of *Salmonella*. While general knowledge of the differences in *Salmonella* virulence gene content has increased over the past decade, specific information on a limited set of virulence genes that could be harnessed for rapid identification of more pathogenic versions of *Salmonella* has been lacking. Here we have described a multiplex PCR assay that targets 8 virulence genes and facilitates the identification of highly pathogenic *Salmonella* (HPS).

*In silico* determination of the presence of these 8 genes in 2,129 closed genome sequences in genbank showed that 7 of the top 10 SoC had an HPSi ≥ 5, with 4 of the top 5 serovars having HPSi = 7 or 8 (Table 3 and Supplementary Table S1). Multiplex PCR assays of 1,303 FSIS isolates showed half had an HPSi ≥ 5, with 97% (635/652) of those identified as SoC. Collectively, 19 serovars on the SoC/StW list had HPSi ≥ 5, and contributed on average 63.4% of the total isolates characterized by CDC each year. Sensitivity and specificity analyses showed the HPS assay has a very strong ability to identify SoC/StW (specificity = 96.3%, sensitivity = 73.5%), with most misclassifications being isolates with HPSi ≤4 that were SoC/StW. There were 13 serotypes in this group which was dominated by Anatum (n = 78), Montevideo (n = 36), Uganda (n = 26), Agona and Senftenberg each with 21 isolates, and Reading (n = 19). This finding highlights a limitation of the HPS assay and suggests these 13 serotypes either possess alternative virulence genes, yet to be identified, that allow them to cause widespread illness and require further investigation, or their presence on the SoC/StW list is due to their increased prevalence or increased contamination levels in food products that are frequently consumed raw, such as fruits and vegetables. In keeping with the latter possibility, serovars Anatum, Montevideo and Agona have been attributed to several large multi-state outbreaks connected with contaminated fruits, seeds, or dry food products such as cereals (Centers for Disease Control and Prevention (CDC), 2010; Miller et al., 2019; Hoffmann et al., 2020; Coulombe and Tamber, 2022). To decrease illnesses attributed to these types of vectors, methods for rapid detection of *Salmonella* contamination levels will be needed, as contamination level and distribution in products that commonly are consumed raw, is likely a greater contributor to illnesses than the presence of a particular cadre of virulence genes.

*Salmonella* SoC with HPSi ≤ 4, that were notably absent or rarely detected in FSIS enrichments were also observed and included serovars Javiana, Oranienburg, Mississippi, Bareilly, Sandiego, Poona, and Paratyphi B. Their absence in the FSIS dataset suggests that food animal production is not a common source of these serovars, and that the risk to public health comes from other sources. Consistent with this notion, these serovars have been associated with outbreaks in fresh produce, dairy products, shell eggs, well-water, and contact with animals (Kumar et al., 2018; Mukherjee et al., 2019; McGeoch et al., 2024; Mitchell et al., 2024). As mentioned above, there is a need for further investigation to identify targets for the rapid detection of these serovars, as collectively they are attributed to 17% of human illness isolates.

Screening new and legacy *Salmonella* WGS data in the NCBI pathogen detection browser for genes that contribute to *Salmonella* pathogenicity in the human host presents the opportunity for predictive modeling and pathogen control (Fenske et al., 2023; Petrin et al., 2023). An example of this was the finding in our *in silico* BLAST analysis, of 87 Enteritidis genome sequences that showed evidence of containing the *sseK3* gene. None of the 109 FSIS Enteritidis strains assayed were observed to amplify this gene. Examining the origin of the strains sequenced showed they were isolated primarily from Asia and Canada (60 and 24%, respectively). Given that *sseK3* has been shown to be carried on GIFSY-1 or ST64B prophage, this may represent a new or emerging lineage of Enteritidis that has acquired a new prophage. Recognizing the value of this *in silico* screening approach for identifying emerging pathogens, NCBI has recently added the genes targeted by the HPS assay to their AMRFinder+ reference gene database [(Feldgarden et al., 2021); https://ftp.ncbi.nlm.nih.gov/pathogen/Antimicrobial_resistance/Data/2024-05-02.2/changes.txt] so that all new *Salmonella* genome sequences in the Pathogen Detection Database^5^ are screened for these virulence genes.

The new approaches being adopted for targeting and identification of NTS have stirred much conversation on how the *Salmonella* scientific community moves forward regarding typing. Do we continue to serotype *Salmonella*? (a legacy typing method with low resolution, high-labor and high-cost that is complicated by the availability of reagents and the effect of genetic drift of the proteins targeted, and by the existence of polyphyletic serovars with different pathogenicities). Or do we adopt a SNP based typing system such as cgMLST to understand *Salmonella* relatedness and conduct traceback investigations (Yoshida et al., 2016; Zhou et al., 2021; Achtman et al., 2022)? Or, given the increased knowledge gained by mining whole genome sequence data of hundreds of thousands of *Salmonella* isolates, do we develop a typing scheme based on the carriage of particular virulence genes (a vgMLST?) to help us rapidly identify SoC or emerging serovars that have acquired new tools, allowing them to expand the niches they occupy and perhaps become better pathogens? At present, there is a tug of war between “old and new” typing methods, and what evolves will likely be a combination that links the two. But regardless of how we proceed with characterizing and typing *Salmonella*, the data presented lay the foundation for molecular assays that will facilitate rapid detection of SoC and improve human health by reducing exposure to this foodborne foe.

## Data Availability

The datasets presented in this study can be found in online repositories. The names of the repository/repositories and accession number(s) can be found in the article/Supplementary material.

## Footnotes

^1^ https://www.fao.org/faolex/results/details/en/c/LEX-FAOC040332/

^2^ https://www.cdc.gov/ncezid/dfwed/BEAM-dashborad.html

^3^ www.geneious.com

^4^ https://www.cdc.gov/ncezid/dfwed/BEAM-dashboard.html

^5^ https://www.ncbi.nlm.nih.gov/pathogens/
