# Supplementary figures and images for: A novel approach for detecting Salmonella enterica strains frequently attributed to human illness—development and validation of the highly pathogenic Salmonella (HPS) multiplex PCR assay
Source: Front Microbiol. 2025 Jan 7;15:1504621. doi: 10.3389/fmicb.2024.1504621 (PMC11752890; doi:10.3389/fmicb.2024.1504621)

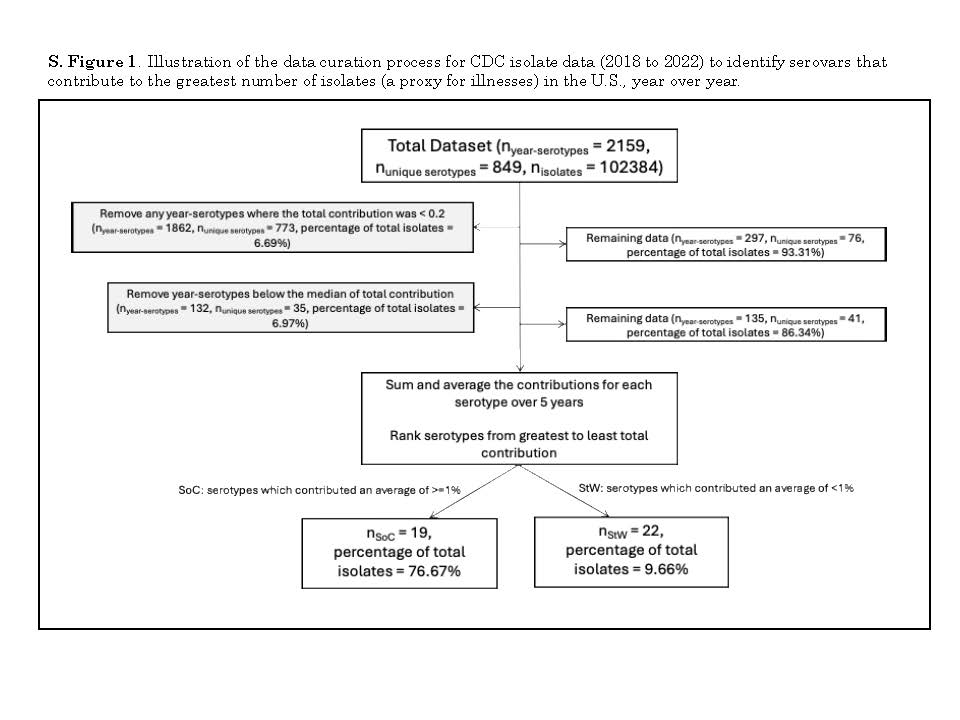

Supplement: Supplementary file 4 [file Image_1.JPEG]
